# The complete mitochondrial genome of *Rhinolophus yunnanensis* (Chiroptera: Vespertilionidae)

**DOI:** 10.1080/23802359.2017.1331323

**Published:** 2017-05-24

**Authors:** Yuanyuan Huang, Leping Zhang, Danna Yu, Kenneth B. Storey, Jiayong Zhang

**Affiliations:** a College of Chemistry and Life Science, Zhejiang Normal University, Jinhua, Zhejiang Province, China;; b Key Lab of Wildlife Biotechnology, Conservation and Utilization of Zhejiang Province, Zhejiang Normal University, Jinhua, Zhejiang Province, China;; c Department of Biology, Carleton University, Ottawa, ON, Canada

**Keywords:** Chiroptera, mitogenome, *Rhinolophus yunnanensis*, phylogenetic relationship, Rhinolophidae

## Abstract

The mitochondrial genome of *Rhinolophus yunnanensis* (Chiroptera: Rhinolophidae) is a circular molecule of 16,865 bp in length with a base composition of 31.2% A, 24.3% T, 29.6% C, 14.9% G. In the control region of *R. yunnanensis*, the sequence of 5'-CAACGTATACACG-3′ repeats 18 times. Phylogenetic analyses indicate that *R. yunnanensis* is a sister clade to ((*Rhinolophus sinicus sinicus* +* R. sinicus sinicus*) + (*R. macrotis* + (*R. pumilu*s + *R. monoceros*))).

Dobson’s horseshoe bats, *Rhinolophus yunnanensis*, are found in China, India, Myanmar and Thailand. Many species of *Rhinolophus* are extremely difficult to distinguish. Although nine complete mitochondrial genomes of *Rhinolophus* are available in GenBank, the complete mitochondrial genome of *R. yunnanensis* has not yet been reported. Hence, in this study, we sequenced the complete mitochondrial genome of *R. yunnanensis* to provide more molecular data to discuss the taxonomic analysis and phylogenetic relationship of *Rhinolophus.*

Samples of *R. yunnanensis* **(**No. GXGL20140711001–GXGL20140711004) were collected from Guangxi, China, and were identified by JY Zhang. The samples were deposited in the lab of Dr. JY Zhang, College of Life Sciences and Chemistry, Zhejiang Normal University. The total genomic DNA **(**No. GXGL20140711001**)** was extracted from liver tissues of *R. yunnanensis* using the Qiagen DNeasy Blood & Tissue Kit (50) (Hilden, Germany). The universal primers for PCR amplification were designed referring to previously published mitochondrial genomes of genera *Myotis* and *Nyctalus* (Kim et al. 2011; Nam et al. 2015; Yoon et al. 2015; Qian et al. 2016; Jiang et al. 2016; Wang et al. 2016; Yu et al. 2016) by primer premier 5.0 (PREMIER Biosoft International, Palo Alto, CA).

The complete mt genome of *R. yunnanensis* contains 22 transfer RNAs genes, 13 protein-coding genes, two ribosomal RNAs and non-coding regions, which is similar to the nine mitochondrial genomes from other *Rhinolophus* species (Nikaido et al. 2001; Lin et al. 2002; Xu et al. 2012; Yoon et al. 2013; Sun et al. 2016; Xie et al. 2016; Zhang et al. 2016; Xiao et al. 2017). The mt genome of *R. yunnanensis* is 16,865 bp in length. The total base composition of *R. yunnanensis* is 31.2% A, 24.3% T, 29.6% C, 14.9% G, with an A + T content of 55.5%. All protein-coding genes begin with ATN as the start codon. *ND2*, *COIII* and *ND4* genes are terminated with an incomplete stop codon T, whereas *ND1* ends with an incomplete stop codon TA, *Cytb* ends with AGA, and the other protein-coding genes end with TAA. The 35-bp sequence between the *tRNA^Asn^* and *tRNA^Cys^* genes is replication origin. The length of the control region of *R. yunnanensis* is 1420 bp with 54.9% AT content, and the sequence 5′-CAACGTATACACG-3′ repeats 18 times.

Phylogenetic relationships were reconstructed using the Bayesian inference (BI) method implemented in MrBayes version 3.1.2 (Huelsenbeck & Ronquist 2001) and maximum likelihood (ML) in RaxmlGUI 1.3 (Silvestro & Michalak, 2012) based on 13 protein-coding genes among 12 species including *Rousettus aegyptiacus* (AB205183 unpublished) and *Pteropus scapulatus* (Lin & Penny 2001) as outgroups. Phylogenetic relationships of BI and ML analyses produced highly concordant topologies (Figure 1). In the BI and ML phylogenetic trees, *R. yunnanensis* is a sister clade to ((*Rhinolophus sinicus sinicus* +* Rhinolophus sinicus sinicus*) + (*Rhinolophus macrotis* + (*Rhinolophus pumilu*s + *Rhinolophus monoceros*))) (1.00 in BI and 82% in ML), *Rhinolophus luctus* is a sister clade to *Rhinolophus formosae* (1.00 in BI and 100% in ML), and (*Rhinolophus ferrumequinum quelpa + Rhinolophus ferrumequinum korai*) is a sister clade to (*R. yunnanensis* + ((*R. sinicus sinicus* +* R. sinicus sinicus*) + (*R. macrotis* + (*R. pumilu*s + *R. monoceros*)))) (0.93 in BI and 64% in ML).

**Figure 1.**
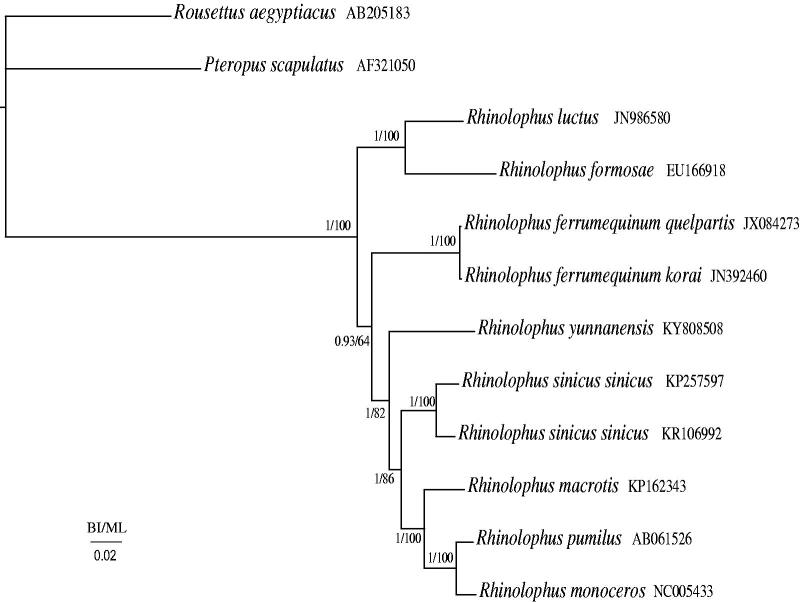
Phylogenetic tree of the relationships among 12 species of Vespertilionidae, including *R. yunnanensis* based on the nucleotide dataset of the 13 mitochondrial protein-coding genes. The Bayesian posterior probability values and the maximum-likelihood bootstrap values are indicated above nodes. The GenBank numbers of all species are shown in the figure.

## Nucleotide sequence accession number

The complete mitochondrial genome of *R. yunnanensis* has been assigned the GenBank accession number KY808508.
